# Effect of exercise training in rats exposed to chronic hypoxia: Application for Monge’s disease

**DOI:** 10.14814/phy2.14750

**Published:** 2021-04-27

**Authors:** José‐Luis Macarlupu, Dominique Marchant, Florine Jeton, Francisco Villafuerte, Jean‐Paul Richalet, Nicolas Voituron

**Affiliations:** ^1^ Laboratorio de Fisiología Comparada Laboratorio de Adaptación a la Altura‐LID Unidad de Transporte de Oxigeno‐IIA Universidad Peruana Cayetano Heredia Lima Peru; ^2^ Laboratoire Hypoxie et Poumon UMR INSERM U1272 Université Sorbonne Paris Nord Bobigny France; ^3^ Laboratory of Excellence GReX Paris France; ^4^ Département STAPS Université Sorbonne Paris Nord Bobigny France

**Keywords:** chronic hypoxia, chronic mountain sickness, exercise training, hemolysis

## Abstract

Physical exercise may improve hematological conditions in high altitude dwellers suffering from Chronic Mountain Sickness (CMS), in reducing hemoglobin concentration. Therefore, the present study aimed to characterize the effects of 1‐month exercise training session in a model of rats exposed to chronic hypoxia. Four groups of male rats were studied: normoxic sedentary (NS, n = 8), normoxic training (NT, n = 8), hypoxic sedentary (HS, n = 8), and hypoxic training group (HT, n = 8). Hypoxic groups were exposed to hypobaric hypoxia for one month (PB =433 Torr). Training intensity was progressively increased from a running speed of 10.4 to 17.8 m/min. Chronic hypoxia led to an increase in hematocrit (HCT) associated with a decrease in plasma volume despite an increase in water intake. Training led to a reduction in HCT (*p* < 0.01), with a non‐significant increase in plasma volume and weight gain. Hypoxia and training had inhibitory effects on haptoglobin (NS group: 379 ± 92; HT: 239 ± 34 µg/ml, *p* < 0.01). Chronic hypoxia and exercise training increased SpO_2_ measured after acute hypoxic exposure. Training blunted the decrease in $\dot{V}$ O_2_ peak, time of exhaustion, and maximum speed associated with chronic exposure to hypoxia. Chronic hypoxia led to a right ventricular hypertrophy, which was not corrected by 1‐month exercise training. Altogether, by decreasing hematocrit, reducing body weight, and limiting performance decrease, training in hypoxia may have a beneficial effect on excessive erythropoiesis in chronic hypoxia. Therefore, regular exercise training might be beneficial to avoid worsening of CMS symptoms in high altitude dwellers and to improve their quality of life.

## Graphical abstract

Exercise training limited chronic hypoxia‐induced decline in physical capacity. Exercise training decreased hematocrit through mechanical hemolysis. Exercise training in hypoxia have a beneficial effect on excessive erythropoiesis

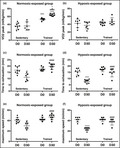

## 1 INTRODUCTION

Permanent living at high altitudes challenges the body to carry out its main functions. As altitude increases, physiological strategies must develop in order to maintain an adequate level of oxygenation at the cellular level. The main adaptations involve respiratory, cardiovascular, and endocrine systems (Bouverot, 1985; Donayre et al., 1968; Gasco et al., 2003; Hainsworth & Drinkhill, 2007; Moncloa et al., 1965, 1966; Monge & Leon‐Velarde, 1991, 2003; Ostadal & Kolar, 2007; Sobrevilla et al., 1967). Within these responses, ventilatory, and hematological acclimatization to chronic hypoxia play a major role. The hematological response consists in an increase in red blood cells and hemoglobin concentration (Hb) secondary to an increase in erythropoietin (Epo) release by the kidneys (Hurtado et al., 1945; Lenfant, 1973; Monge & Whittembury, 1976; Siques et al., 2006, 2007). In parallel, a hypoxia‐induced decrease in plasma volume also contributes to the increase in Hb concentration (Richalet et al., 1983). In spite of these physiological adaptations, exercise performance, as measured by maximal O_2_ consumption (V̇O_2_ max) decreases at high altitude (Fulco et al., 1998).

The loss of adaptation, or the inability to adapt, leads to Chronic Mountain Sickness (CMS), also known as Monge's disease (Monge & Leon‐Velarde, 1991; Villafuerte & Corante, 2016). This is a serious illness characterized by a combination of various clinical symptoms, associated with excessive erythrocytosis (EE), arterial hypoxemia, sometimes hypoxia associated pulmonary hypertension and, in the last stages of the illness, cardiac failure (Villafuerte & Corante, 2016). Current treatments consist mainly in non‐pharmacologic (bloodletting) or pharmacologic (acetazolamide, methylxantine,…) reduction of EE (Leon‐Velarde et al., 2005; Richalet et al., 2008; Rivera‐Ch et al., 2007; Villafuerte & Corante, 2016). Descent to low altitude is efficient but has many psychological, familial and socio‐economical adverse consequences. Therefore, there is currently no absolutely safe and efficient treatment for CMS (Rivera‐Ch et al., 2007).

The tolerance of these patients to exercise remains controversial. Some studies showed that CMS patients present a limited exercise tolerance (Leon‐Velarde et al., 2005; Pratali et al., 2012) probably due to interstitial lung water accumulation at exercise (Pratali et al., 2012). It is well‐established that exercise increases pulmonary artery pressure (Leavitt et al., 1991) and may induce an exaggerated increase in pulmonary arterial pressure in patients with pulmonary pre‐hypertension (Mininni et al., 1996). However, it seems that exercise‐induced pulmonary hypertension in CMS patients does not affect their exercise capacity (Naeije & Vanderpool, 2013). Indeed, the aerobic exercise capacity of CMS patients seems to be preserved despite severe pulmonary hypertension and relative hypoventilation (Groepenhoff et al., 2012). In a model of rats exposed to chronic hypoxia, our team showed that exercise improved lung gas exchange and attenuated acute hypoxic pulmonary hypertension but did not prevent chronic hypoxia‐induced pulmonary hypertension (Favret et al., 2006). Furthermore, exercise training in hypoxia attenuates myocardial adrenoceptor downregulation and preserves cardiac output (Q̇c) and maximal O_2_ consumption (V̇O_2_ max) after acclimatization. This finding supports the hypothesis that β‐adrenoceptor downregulation partially contributes to the limitation of V̇O_2_max after acclimatization in rats (Favret et al., 2003). As Vascular Endothelial Growth Factor (VEGF) gene expression, an essential determinant of skeletal muscle angiogenesis is enhanced in hypoxia associated with exercise training, this could also play a role in preservation of muscle performance in chronic hypoxia (Breen et al., 2008).

Developing physical activity in CMS patients might be of interest in the context of a non‐pharmacological therapeutic approach to ameliorate health status of these patients and to control the progression of symptoms. It was proposed that addressing risk and aggravating factors of CMS would be a potentially beneficial preventive approach to reduce symptoms and ameliorate the quality of life of CMS patients (Rivera‐Ch et al., 2007; Villafuerte & Corante, 2016). Therefore, this project aimed to characterize the effects of 1‐month exercise training session in a model of rats exposed to chronic hypoxia with a particular emphasis on metabolic variables, hematological status, and cardiac hypertrophy.

We hypothesized that exercise training may decrease HCT through an increased hemolysis due to repeated impacts during running. As a decrease in haptoglobin is known to reflect hemolysis, we measured plasma haptoglobin concentration as a marker of a possible hemolysis.

## 2 METHODS

### 2.1 Ethical approval

Experimental protocols were approved by the Ethics Committee for Animal Experiment Charles Darwin and the French ministry of research (APAFIS#11051‐201708282253473 v2), done in accordance with the European Communities Council Directive of September 22, 2010 (2010/63/EU) for animal care, and conducted in accordance with the French legislation for animal care.

### 2.2 Animals

As CMS is largely a male disease, our study was conducted only in males. Fifty‐six male Sprague Dawley rats (aged 6 to 8 weeks, body weights in Table 1) were hosted in normoxic or hypoxic conditions and were trained or not. Thus, four groups have been formed: normoxic sedentary group (NS, n = 8), normoxic training group (NT, n = 8), hypoxic sedentary group (HS, n = 8), and hypoxic training group (HT, n = 8). The hypoxic‐exposed groups (HT and HS) were exposed to hypobaric hypoxia for one month (PB = 433 Torr; P_i_O_2_ = 90.9 Torr, simulated altitude ≈ 4500 m). Hypoxia was maintained by a vacuum source at flow rates sufficient to prevent CO_2_ build‐up (fraction of inspired CO_2_<0.003). All animals were housed in a 12 h/12 h light/dark cycles at an ambient temperature of 20–22°C. The chamber was returned to sea level (756 Torr) for 30 minutes each day of training for animal husbandry. A pellet diet and water was provided ad libitum. Mean food and water intake were measured daily.

**TABLE 1:** Primers used for real‐time polymerase chain reaction

GENE	Forward Primer	Reverse Primer
VEGF (98 bp)	5′‐CTGGACCCTGGCTTTACTGC−3′	5′‐ACTTCACCACTTCATGGGCTT−3′
18S (160 bp)	5′‐GTAAGTGCGGGCCATAAGCTT−3′	5′‐AGTCAAGTTCGACCGTCTTCTCA−3′

Five days a week (Monday to Friday), in the morning, HT and NT groups carried out 1 h of exercise training protocols in hypoxic or in normoxic conditions respectively.

Animals were shared out in two sets of protocols. Thus, 32 animals were used to evaluate the metabolic parameters, hematocrit, and blood cell count at the beginning (D0, PRE values) and after one month (D30, POST values). Thereafter, animals were euthanatized for tissues sampling. For the second sets of experiments, 24 animals were used to evaluate saturation and heart rate at D0 and D30 and were then euthanatized to evaluate blood and plasma volumes, Fulton's ratio, and vascular endothelial growth factor (VEGF) level in the gastrocnemius and soleus muscles.

### 2.3 Exercise training protocols

The training of the animals was performed on a six‐lane treadmill (1 animal/lane) designed for the training of rats (2.5 meters long by 1.5 meters wide, inclination: 10%; Treadmill Manutan Bonfiglioti Components Italy). During training sessions, rats breathe either normoxic gas (NT group) or hypoxic gas (HT group, F_i_O_2_ = 0.12). For this, the treadmill was positioned in a chamber where a normoxic or a hypoxic gas were flushed. The NT and HT groups performed a physical training 1 h per day, 5 days per week (Monday to Friday). During this time the HS group remained in hypoxic conditions. Before each training session, each rat was weighed and placed in its respective lane inside the treadmill. The training intensity was progressively increased and conducted as follows: week 1, running speed = 10.4 m/min; week 2, running speed = 13.1 m/min; week 3, running speed = 14.8 m/min; week 4, running speed = 16.8 m/min. No electrical shocks or aversive stimuli were used during training sessions, so as to encourage rats to run. At the end of the training session, the animals were removed and returned to their respective cages with water and food ad‐libitum.

### 2.4 Metabolic variables

The measurement of the metabolic variables was made using a specific treadmill (treadmill Simplex II. Columbus Instruments) designed for the recording of the oxygen consumption (V̇O_2_) and carbon dioxide output (V̇CO_2_). The treadmill was ventilated with an airflow inlet at 2.5 L/min. Instantaneous fraction of O_2_ and CO_2_ were evaluated continuously at the inflow and outflow with gases’ analyser for O_2_ and CO_2_ (FC‐10 and CA‐10 respectively; Sable system, Las Vegas, USA) connected to a data acquisition system. The airflow was desiccated before entering in the analyser. V̇O_2_ was calculated as previously described (Jeton et al., 2017; Marcouiller et al., 2014) according to the following formula and was normalized by the body weight:

$$\overset{\cdot }{V}O_{2}=\;\text{flow}\times \left(\left(F_{i}O_{2}‐F_{e}O_{2}\right)‐F_{e}O_{2}x\left(F_{e}{\text{CO}}_{2}‐F_{i}{\text{CO}}_{2}\right)\right)/\left(1‐F_{e}O_{2}\right)$$

where F_i_ and F_e_ are the fraction of O_2_ and CO_2_ in the inflowing and outflowing lines respectively.

Before the start of each test, the animals were weighed and then warmed‐up on the treadmill at a minimum speed of 10 m/min for 3 minutes. After that, the V̇O_2_ peak test started at 10 m/min and speed was then increased by 4 m/min every 2 min. The test was stopped when the animal could no longer run and preferred to stay still at the bottom of the treadmill despite delivery of a mild electrical shock. The intensity of electrical shock was adjusted to 1.5 mA and the protocol was quickly stopped after 2 consecutive shocks. At this time, V̇O_2_ peak (the highest value of V̇O_2_ attained during the test, ml/min/kg), time to exhaustion (the maximum duration of the test, min), and maximum speed (the maximal speed reached during the test, m/min) has been recorded.

### 2.5 Hematocrit and blood cell count

After deep isoflurane anesthesia (induction at 1 L/min, 5% isoflurane, 5 min and then face mask at 200 ml/min, 2% isoflurane), blood samples were collected at the tail level before and after one month of training and or hypoxia protocol (or equivalent time for NS groups). A volume of 70 µl was collected for hematocrit (HCT) and 300 µl for blood cell count.

### 2.6 Blood and tissue sampling

After lethal IP injection of pentobarbital (100 mg/Kg), rats were exsanguinated and blood was collected (Around 2 ml). Blood was then centrifuged (800 G, 10 min) and plasma was collected. Then rib cage was opened and the heart was removed. After atria suppression, right ventricle (RV) and left ventricle +septum (LV + S) was isolated and weighted in order to determinate Fulton's ratio (RV/LV + S), an index of right ventricle hypertrophy. Finally, the *soleus* (SO; oxidative) and the *gastrocnemius* (GA; glycolytic) muscles (left side) were sampled for molecular biology. For this, the rear tendon was cut distally, underneath the heel. After that, the posterior muscle was pulled‐up and back‐warded. In this position, the tendon of the SO become visible and can be cut and removed. Finally, the GA was removed by holding it from the distal tendon and by cutting at the proximal end.

### 2.7 Peripheral oxygen saturation and heart rate

Oxygen saturation and heart rate were assessed via a non‐invasive method of infrared pulse oximetry (mouseOx Plus, Starr life science) (Gille et al., 2018; Lax et al., 2014). After short sedation, animals’ neck was shaved and then the collar sensor was put in place. After waking, rats were habituated for 10 min and the peripheral blood saturation (SpO_2_, %) and heart rate (HR, bpm) were recorded during 10 min in normoxic condition (F_i_O_2_ 0.21) and 10 min in normobaric acute hypoxic condition (F_i_O_2_ 0.12) in normoxic and hypoxic‐exposed groups. These measurements were performed before (D0) and after the chronic exposure to hypoxia or normoxia (D30).

### 2.8 Blood and plasma volumes

Separate sets of rats were used to measure plasma volume via a modified Evans blue dilution method. As previously described (Richalet et al., 2018), animals were anesthetized with intra‐peritoneal pentobarbital injection (60 mg/kg) and HCT (%) was measured via blood sampling from the tail (70 µl). Then, a catheter was inserted in the right jugular vein for Evans blue injection and another one in the left carotid artery for blood sampling (Feng et al., 2015). After initial blood sampling, Evans blue (200 mg in 200 µl of saline) was injected slowly and the catheter was then flushed with 350 µl of saline. Then blood was sampled (700 ± 50 µl) at 2, 4, and 6 min after the end of injection. Plasma volume was calculated via a two‐compartment model‐based method (Richalet et al., 2018). Total blood volume was calculated from plasma volume and hematocrit.

### 2.9 Plasma haptoglobin, soluble Epo‐receptor, and soluble transferrin‐receptor

The concentration of haptoglobin, soluble Epo‐receptor (sEpoR), and soluble transferrin receptor (sTfR) was detected in plasma with ELISA approach. All the tests were done according to manufacturer recommendations. Kits used were, respectively, for Haptoglobin, soluble EPO receptor, and soluble transferrin receptor: rat Haptoglobin: CSB‐E08587r96T, Cusabio; rat soluble Epo receptor, SEA027Ra‐96, Cloud Clone Corp; rat soluble transferrin receptor, MBS268897‐96, MyBioSources.

### 2.10 RNA extraction and reverse transcription‐quantitative polymerase chain reaction of vascular endothelial growth factor (VEGF)

Left *Gastrocnemius* and *soleus* muscles were collected after in vivo experiments: 50 to 100 mg of each rat muscle were homogenized in TRIzol™ Reagent (Qiagen, Courtaboeuf, France) using an Ultra‐Turrax® system, following manufacturer's instructions. Extracted ARN were re‐suspended in 50 µl DEPC H2O and quantified using a BioSpec‐Nano spectrophotometer (Life Science, Shimadzu, Marne‐la‐Vallée, France) at 260 nm. Single‐strand cDNAs were synthesized from 1 µg of total RNA using maxima first strand cDNA synthesis kit composed by a mixture of oligo (dT) and random hexamer primers according to the manufacturer's instructions (Thermo Fisher, Illkirch cedex, France). Resulting cDNA samples were amplified by quantitative polymerase chain reaction (PCR) with Absolute qPCR SybrGreen Rox mix (Thermo Fisher, Illkirch cedex, France) on StepOne Plus Real‐Time PCR system (Applied Biosystem, Life technology, Thermo Fisher, Illkirch cedex, France). Length and sequence of primers used for quantitative real‐time PCR are described in Table 1. All samples were performed in duplicate. Cycle threshold values were normalized to the amplification of the endogenous reference gene 18S for each transcript. The expression levels were calculated using the ratio:

$$\frac{\text{Experimental condition}}{\text{Control condition}}\;\;=\;\frac{{\left(\text{Amplification efficiency}\;18S\right)}^{\Delta \text{Ct}\;18S}}{{\left(\text{Amplification efficiency}\;\text{VEGF}\right)}^{\Delta \text{Ct}\;\text{VEGF}}}$$

Fold increase expression were reported to normoxic sedentary condition.

### 2.11 Statistical analysis

Data were expressed as mean ± standard deviation. D’Agostino‐Pearson omnibus normality test was realized to assess the distribution of the data. All variables were analysed using a two‐way ANOVA followed by Tukey test for multiple comparisons. Furthermore, haptoglobin comparison was forced by using unpaired t‐test. All analyses were performed with the Graph Pad – Prism software (Graph Pad software, La Jolla, CA, USA), and differences were considered significant when *p* < 0.05.

## 3 RESULTS

### 3.1 Body weight and eating behavior

Over one month, all groups of rats had gained weight (Table 2). Chronic hypoxia led to an increase in water intake but a decrease in food intake leading to a lower weight gain as compared to normoxia‐exposed groups. Training led to a decrease in food intake leading to a lower weight gain as compared to sedentary groups. As expected, animals of the NS group were those who gained most weight.

**TABLE 2:** Body weight, hematocrit, plasma and blood volume, mean water and food intake.

	ANOVA main effects	Normoxic‐exposed group		Hypoxic‐exposed group	
		Sedentary NS	Trained NT	Sedentary HS	Trained HT
Initial body weight (g)	ns	364 ± 77	339 ± 30	282 ± 46	326 ± 7
Weight gain (g)	**,###	285 ± 105	88 ± 34^###^	142 ± 25**	74 ± 17###
Hematocrit (%)	***,##	48.4 ± 2.1	44.9 ± 2.1^#^	60.1 ± 2.6***	57.7 ± 2.7***
Plasma volume (mL/kg)	*	34.6 ± 9.4	39.7 ± 11.1	25.1 ± 6.1*	26.8 ± 11.5*
Blood volume (mL /kg)	ns	66.7 ± 16.1	72.9 ± 20.9	62.6 ± 12.4	64.7 ± 30.8
Water intake (mL/d)	***	31.0 ± 2.4	32.9 ± 4.0	41.4 ± 10.1***	41.9 ± 10.6***
Food intake (g/d)	***,###	23.2 ± 2.4	20.3 ± 0.8^###^	21.0 ± 2.4***	18.0 ± 2.1***,###

Values are mean ± standard deviation.

Hypoxia vs. Normoxia: *p < 0.05, **p < 0.01, ***p < 0.001.

Trained vs. Sedentary: # *p* < 0.05, ## *p* < 0.01, ###*p* < 0.001.

### 3.2 Hematocrit, plasma volume, and peripheral oxygen saturation

Chronic hypoxia induced an increase in HCT associated with a decrease in plasma volume (Table 2), despite an increase in water intake as mentioned above. Training led to a reduction in HCT with a tendency to an increase in plasma (*ns*) (Table 2).

**FIGURE 1:**
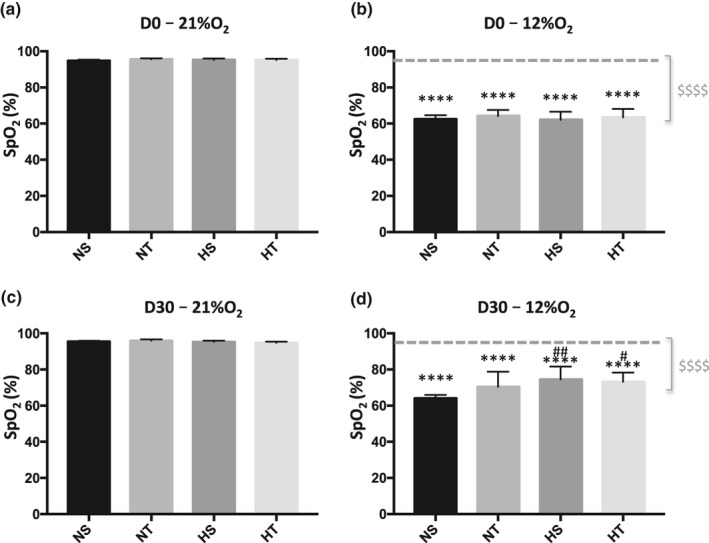
Peripheral oxygen saturation (SpO_2_) at 21% of O_2_ (a and c) and 12% O_2_ (b and d) at the beginning of experiments (D0, a and b) and after one month (D30, c and d). Dashed grey line (b and d) indicates the mean of SpO_2_ at 21% O_2_. $ in grey indicates global effect of acute hypoxia ($$$$, *p* < 0.0001). * indicates significant differences between SpO2 at 21% and 12%O_2_ at D0 and D30 (****, *p* < 0.0001). # indicates significant differences between pre and post‐condition (#, *p* < 0.04; ##, *p* < 0.003). NS, Sedentary Normoxic‐exposed group; NT, Trained Normoxic‐exposed group; HS, Sedentary Hypoxic‐exposed group; HT, Trained Hypoxic‐exposed group

At D0 and D30, acute hypoxia elicited a significant decrease in SpO_2_ in all groups (Figure 1). No difference was observed at 21% O_2_ between groups exposed for one month to normoxia or hypoxia (Figure 1a,c). However, hypoxic‐exposed groups displayed a less important oxygen desaturation during acute hypoxia (Figure 1b,d).

### 3.3 Plasma concentration of haptoglobin, soluble Epo‐receptor, and soluble transferrin‐receptor

We did not observe any significant effect of hypoxia or training on plasma concentration of sEpoR and sTfR (Table 3). Two‐way ANOVA revealed a main hypoxic effect on haptoglobin concentration (*p* < 0.001) and a tendency for a training effect (*p* = 0.059). Indeed, plasma haptoglobin was lower in the HT compared to the NT group (Table 3). The haptoglobin concentration seems to be lower in the HT compared to HS group, but Two‐way ANOVA results are not significant. However, when comparison was artificially forced using unpaired t‐test, we observed a significantly lower haptoglobin concentration in HT vs HS group (*p* < 0.01).

**TABLE 3:** Plasma haptoglobin, soluble erythropoietin receptors (sEpoR), and soluble transferrin receptors (sTfR) in sedentary and trained rats in Normoxia and Hypoxia‐exposed groups

	ANOVA main effects	Normoxic‐exposed group		Hypoxic‐exposed group	
		Sedentary NS	Trained NT	Sedentary HS	Trained HT
Haptoglobin (µg/ml)	***,&	380 ± 92	355 ± 72	311 ± 68***	239 ± 34***
sEpoR (pg/ml)	ns	4239 ± 868	4404 ± 1045	3955 ± 669	4703 ± 1246
sTfR (µg/ml)	ns	1630 ± 132	1608 ± 497	1450 ± 265	1694 ± 45

Values are mean ± standard deviation.

Hypoxia vs. Normoxia: ***p < 0.001 ,

Trained vs. Sedentary: & *p* = 0.059.

### 3.4 Vascular endothelial growth factor (VEGF) level in the gastrocnemius and soleus muscles

**FIGURE 2:**
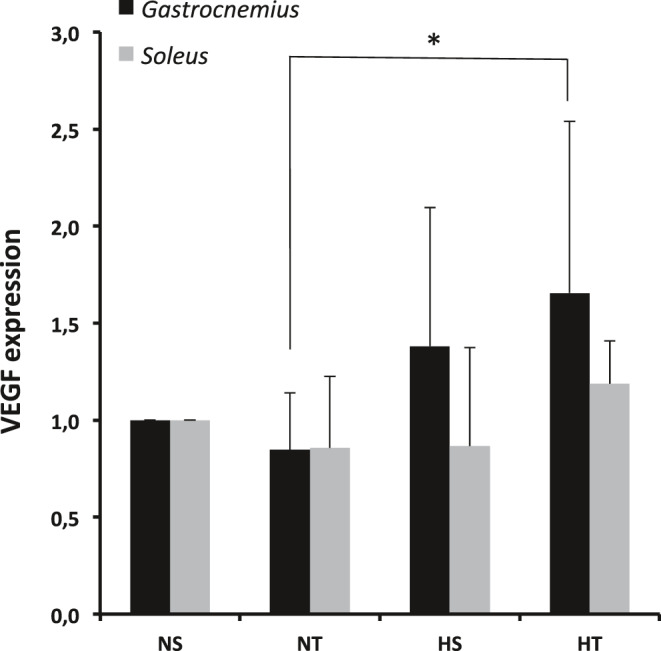
Expression of Vascular Endothelial Growth Factor (VEGF) in *Gastrocnemius* and *Soleus* muscle. Significant global effect of hypoxia in the *Gastrocnemius* (*p* = 0.01). *: *p* < 0.05 HT vs NT. NS, Sedentary Normoxic‐exposed group; NT, Trained Normoxic‐exposed group; HS, Sedentary Hypoxic‐exposed group; HT, Trained Hypoxic‐exposed group

Neither hypoxia nor training had an effect on the expression of VEGF in the soleus muscle in our experimental conditions. Conversely, hypoxia increased VEGF expression in the gastrocnemius (*p* < 0.05). Training had no significant effect (Figure 2).

### 3.5 Cardiac characteristics

**FIGURE 3:**
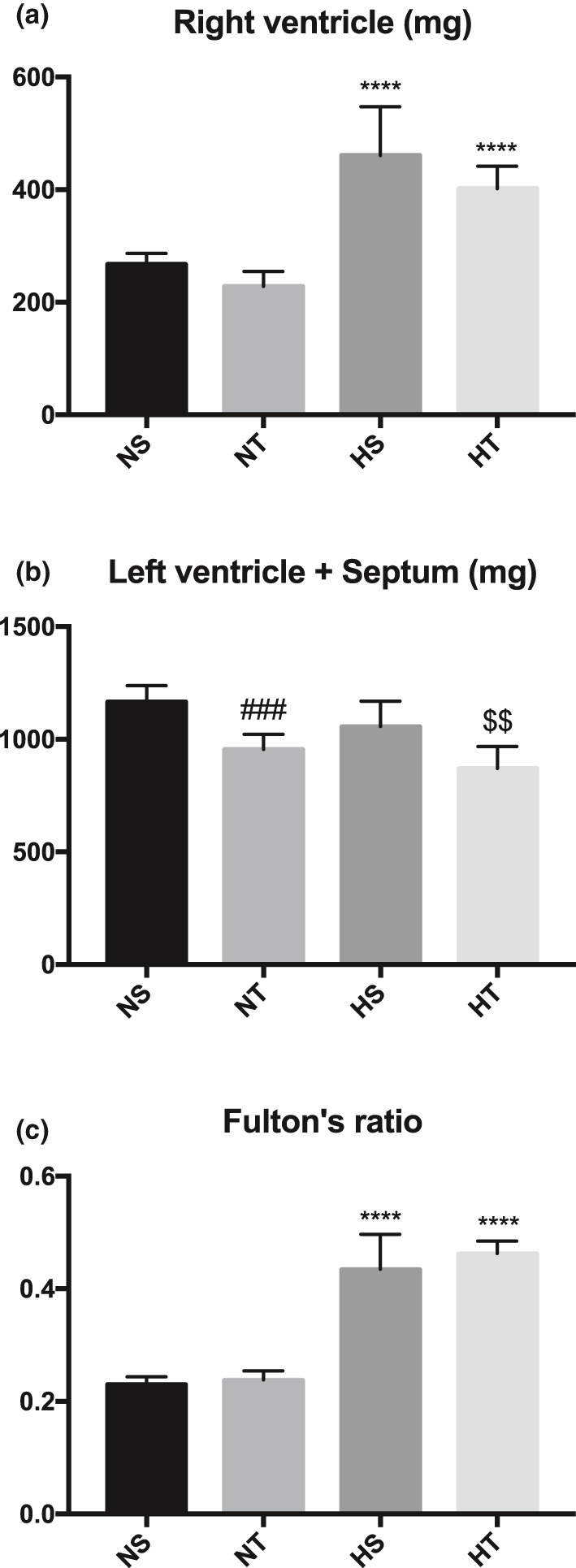
Weight of right ventricle (a), left ventricle + septum (b) and Fulton's ratio (c) in sedentary and trained rats exposed or not to chronic hypoxia. * indicates significant differences between normoxic and hypoxic‐exposed rats (****, *p* < 0.0001). # and $ indicates significant differences between sedentary and trained rats in normoxic and hypoxic‐exposed group respectively (###, *p* < 0.0008; $$, *p* < 0.002). NS, Sedentary Normoxic‐exposed group; NT, Trained Normoxic‐exposed group; HS, Sedentary Hypoxic‐exposed group; HT, Trained Hypoxic‐exposed group

Hypoxia led to an increase in Fulton's ratio mainly due to an increase in right ventricular weight (Figure 3c). Training slightly decreased left ventricular weight (Figure 3b) but did not reverse right ventricular hypertrophy (Figure 3a).

**FIGURE 4:**
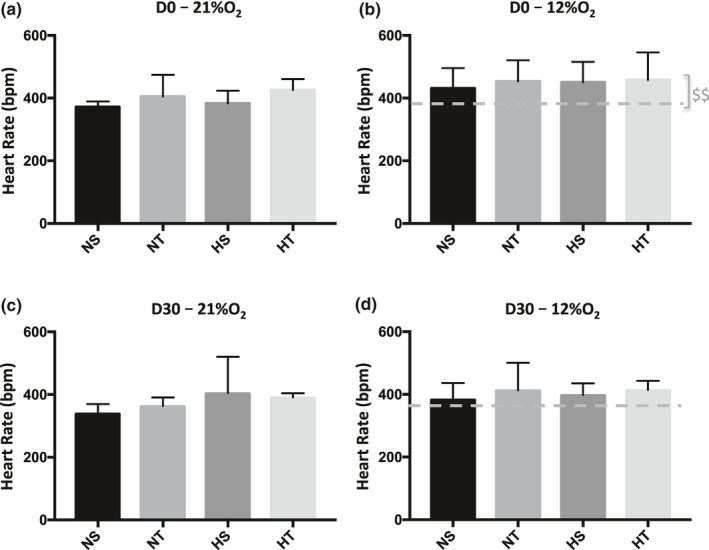
Heart rate at 21% of O_2_ (a and c) and 12% O_2_ (b and d) at the beginning of experiments (D0, a and b) and after one month (D30, c and d). Dashed grey line indicates the mean of heart rate at 21% O_2_._._ $ in grey (b) indicates global effect of acute hypoxia (21% O_2_ vs 12% O_2_; $$, *p* < 0.006). NS, Sedentary Normoxic‐exposed group; NT, Trained Normoxic‐exposed group; HS, Sedentary Hypoxic‐exposed group; HT, Trained Hypoxic‐exposed group

We observed a slight, but significant effect of acute hypoxia on heart rate at D0 (before exposure) (Figure 4a,b) while this effect was blunted at D30 (after exposure) (Figure 4c,d). Heart rate was not modified after chronic hypoxia (Figure 4a,c). Finally, no effect of training and no interaction between hypoxia and training were observed on heart rate.

### 3.6 Metabolic variables during maximal exercise

**FIGURE 5:**
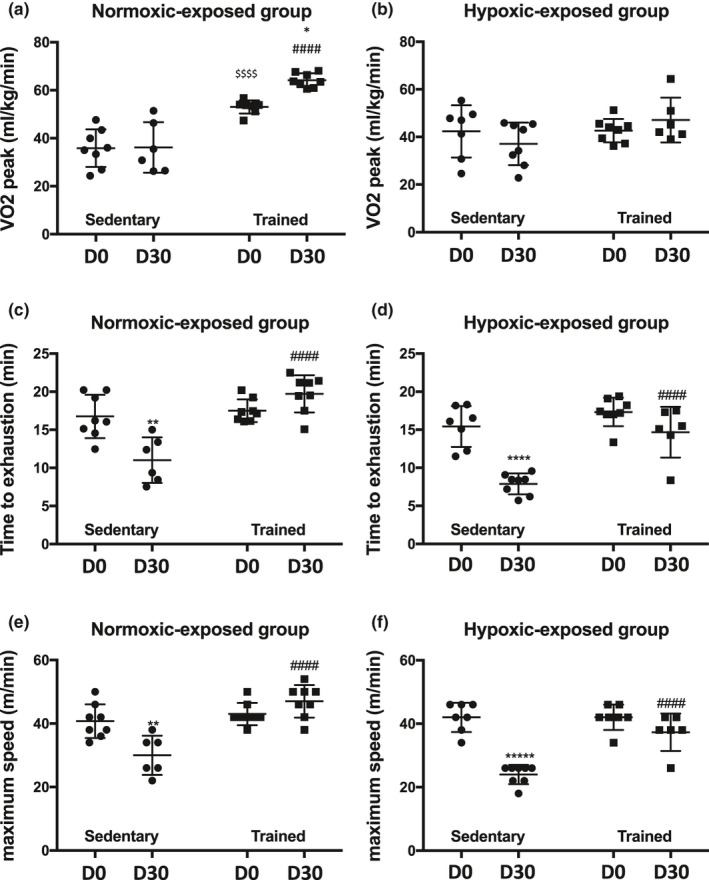
Metabolic variables in normoxia‐exposed groups (a, c, e) and hypoxia‐exposed groups (b, d, f) for sedentary or trained rats at the beginning of the experiment (D0) and after one month (D30). $ indicates a significant difference between sedentary and trained group at D0 ($$$$, *p* < 0,001). * indicates comparison between D0 and D30 in sedentary or trained group (****, *p* < 0.0001; **, *p* < 0.003). # indicates comparison between sedentary and trained group at D30 (####, *p* < 0.0001)

As expected, one month of training enhanced V̇O_2_ peak, time to exhaustion, and maximum speed (Figure 5a,c,e), while a decrease in time to exhaustion and maximum speed was observed in sedentary rats (Figure 5c,e).

Chronic hypoxia led to a decrease in time to exhaustion and maximum speed in sedentary rats (Figure 5D and 5F). However, in the hypoxic‐exposed groups, trained rats displayed a higher time to exhaustion and maximum speed than sedentary animals (Figure 5d,f).

## 4 DISCUSSION

Chronic hypoxia induced an increase in hematocrit (HCT) associated with a decrease in plasma volume despite an increase in water intake, while a reduction in food intake and weight gain was observed. Training led to a reduction in HCT, food intake, and weight gain, with a non‐significant increase in plasma volume. Hypoxia and training had inhibitory effects on haptoglobin. Chronic hypoxia and exercise training blunted the decrease in SpO_2_ induced by acute hypoxic exposure. However, there was no cumulative effect of both. Chronic hypoxia blunted the increase in heart rate induced by acute hypoxia, confirming the downregulation of the adrenergic system in chronic hypoxia (Favret et al., 2001). However, no effect of training was observed on this mechanism. Training blunted the decrease in time to exhaustion and maximum speed associated with chronic exposure to hypoxia. However, after one month, sedentary rats were not re‐acclimatized to the treadmill, so we cannot exclude that this point was linked, at least in part, to the reduction in performance. Furthermore, we cannot exclude that the trained groups were mastered and felt comfortable on the treadmill as compared to sedentary groups.

Chronic hypoxia led to right ventricular hypertrophy, which was not corrected by 1‐month exercise training. Nevertheless, we suggest that by decreasing hematocrit, exercise training in chronic hypoxia may have a beneficial effect on excessive erythropoiesis.

### 4.1 Exercise training increased hemolysis and led to a lower hematocrit

Our results suggest that exercise led to a reduction in HCT without significant modification of plasma volume or total blood volume. This is in accordance with previous studies showing that training decreases HCT. Indeed, in trained subjects living above 4000 m, HCT was lower than in sedentary ones living at the same altitude (48% vs. 54% respectively) (Cornolo et al., 2005). Furthermore, our results showed that hypoxia and training could induce a decrease in plasma haptoglobin. The ELISA kit we used detects total haptoglobin. Thus, as we observed a decrease in haptoglobin, this suggests that unbound haptoglobin decreased, due to an increase in the binding between haptoglobin and hemoglobin (Cooper et al., 2013; Hwang & Greer, 1980). The formation of this complex is fast, quickly eliminated from plasma and then degraded by the macrophages of the spleen, liver, and bone marrow (Schaer et al., 2006).

It was previously suggested that physical activity may lead to the rupture of red blood cells (RBC) due to mechanical events, osmotic, or oxidative stress (Bonilla et al., 2005; Bula et al., 1966; Schobersberger et al., 1990; Selby & Eichner, 1986; Telford et al., 2003). As this effect is due to the passage of RBC into foot capillaries during foot strike (Telford et al., 2003), it is expected to be more important in impact sports such as running. However, in our knowledge, there is also evidence of hemolysis induced by exercise in disciplines where the impact is very small or is not present (Bula et al., 1966; Lippi & Sanchis‐Gomar, 2019; Schobersberger et al., 1990; Selby & Eichner, 1986; Telford et al., 2003). The hemolysis rate would occur to a greater degree when the blood shows a higher viscosity, as is the case of animals exposed to chronic hypoxia. Therefore, this group of animals is submitted to a mechanical effect (repeated impacts for running) and the effect of a higher blood viscosity, leading to an additive effect that would favor hemolysis, as shown by the decrease in plasma haptoglobin.

### 4.2 Exercise training reduced chronic hypoxia‐induced decline in physical capacity

High altitude is associated with a decline in physical performance (Brouns, 1992; Chaudhary et al., 2012; Sridharan et al., 1987). Although exercise induces an increase in pulmonary arterial pressure in patients (Mininni et al., 1996), it seems that exercise does not affect exercise capacity despite severe pulmonary hypertension and relative hypoventilation (Groepenhoff et al., 2012; Naeije & Vanderpool, 2013). This paradoxical effect might be due to high O_2_ carrying capacity of blood and increase in lung diffusing capacity (Groepenhoff et al., 2012; Naeije & Vanderpool, 2013).

Other studies have shown that physical training in hypobaric or normobaric hypoxia generates a greater vasodilatory response in lung tissue that is useful for preventing hypoxia‐induced pulmonary arterial hypertension. Indeed exercise could be useful in preventing pulmonary hypertension induced by hypoxia and exercise (Kashimura & Sakai, 1991). Therefore, as shown by our results, 5 h/week moderate exercise training reduced the decline in physical capacity in rats chronically exposed to hypoxia. These results open an interesting option to ameliorate health status in CMS patients in addition to pharmacological treatments. The expression of VEGF gene was enhanced in hypoxic conditions in the gastrocnemius muscle, as previously shown in mice (Hagström et al., 2010), but there was no significant additive effect of training, conversely to what was shown by others (Olfert et al., 2001). Interestingly, a specific VEGF genotype was found associated with Chronic Mountain Sickness in Peruvian high altitude dwellers (Espinoza et al., 2014).

Importantly, exercise training limited the weight gain of our rats. As obesity increases the risk of developing CMS and the severity of the condition (Gazal et al., 2019; Rivera‐Ch et al., 2007), limiting weight increase is of particular interest. Conversely, in sedentary rats, we observed after one month a decrease in time to exhaustion and in maximum speed, probably due to the increase in body weight.

These observations have to be confirmed in humans in order to propose exercise training as an efficient tool to control excessive erythrocytosis in CMS patients.

In conclusion, the present study suggests that exercise training in chronically exposed rats have beneficial effects by preventing the decrease in exercise performance, by reducing body weight and by improving hematocrit. As CMS is characterized by slow, but irreversible, aggravation of symptoms and quality of life, it is important to find means to slow down this unfavorable development. On the basis of our results, we suggest that regular exercise training might be beneficial to avoid worsening of CMS symptoms in high altitude dwellers.

## CONFLICT OF INTEREST

We have no conflict to interest to disclose.

## AUTHOR CONTRIBUTIONS

J.P.R. and N.V. designed the experiments. J.L.M., D.M., F.J., J.P.R., and N.V. performed, analysed, and interpreted experiments. J.P.R. and N.V. wrote the manuscript. J.L.M., D.M., F.J., and F.V. revised the manuscript for important intellectual content. All authors approved the final version of the manuscript, agreed to be accountable for all aspects of the work and consented to its submission to physiological reports as a research. All persons designated as authors qualify for authorship, and all those who qualify for authorship are listed.
